# Spontaneous Recovery from a Chronic Prolapsus Penis in the Horse

**Published:** 1898-06

**Authors:** 


Spontaneous Recovery from a Chronic Prolapsus Penis in the Horse.—District veterinarian Röder reports that the purchaser of a five-year-old Wallachian horse noticed immediately after his purchase, in March, 1892, that the penis hung out of its sheath almost at arm's length, both while walking and trotting. The cause of the trouble could not be ascertained, and all treatment was futile. After continuing about five years the trouble began to improve spontaneously, so that the projection did not exceed a hand's breadth.—*Idem.*

